# Draft Genome Sequence of *Dicyma pulvinata* Strain 414-3, a Mycoparasite of *Cladosporium fulvum*, Causal Agent of Tomato Leaf Mold

**DOI:** 10.1128/MRA.00655-19

**Published:** 2019-08-29

**Authors:** Hirotoshi Sushida, Takuya Sumita, Yumiko Higashi, Yuichiro Iida

**Affiliations:** a National Agriculture and Food Research Organization, Tsu, Mie, Japan; Vanderbilt University

## Abstract

Dicyma pulvinata strain 414-3, isolated from the surface of a tomato leaf, is a mycoparasitic fungus of Cladosporium fulvum, which causes leaf mold of tomato. We report here the draft genome sequence of strain 414-3, which will contribute to elucidating the molecular mechanisms involved in the mycoparasitism.

## ANNOUNCEMENT

Leaf mold caused by Cladosporium fulvum Cooke (syn. Fulvia fulva, Passalora fulva) is one of the major diseases of tomato. The fungus enters the host through stomata, colonizes the apoplastic region between mesophyll cells, and emerges from stomata and causes discolored patches on the leaves. Twelve physiological races have now evolved that overcome all the resistant tomato cultivars commercially developed in Japan (1). In addition, fungicide-tolerant strains have also been reported, so an integrated pest management (IPM) strategy needs to be devised and should include a biocontrol agent(s) rather than fungicides.

Our group previously isolated Dicyma pulvinata strain 414-3 because it was growing over *C*. *fulvum* lesions on a tomato leaf and seemed to prevent spread of the fungus and disease in the greenhouse (2). Strain 414-3 parasitizes *C. fulvum* independent of races but not pathogens that cause other foliar tomato diseases. Although *D*. *pulvinata* produces the antifungal sesquiterpene deoxyphomenone, which inhibits growth of *C. fulvum* (3, 4), the molecular basis underlying fungal-fungal recognition mechanisms and the host range of the mycoparasite are not fully known. Thus, to identify genes that are related to the mycoparasitism of *D*. *pulvinata* strain 414-3, we sequenced its genome, and here, we report its draft genome sequence.

Strain 414-3 was grown in potato dextrose broth (BD Difco, Franklin Lakes, NJ, USA), and the mycelium was ground in liquid nitrogen to isolate genomic DNA using a standard protocol with phenol-chloroform extraction and ethanol precipitation (2). DNA libraries were prepared using a TruSeq Nano DNA low-throughput library prep kit (Illumina, Hayward, CA, USA) and a DNA template preparation kit (Pacific Biosciences, Menlo Park, CA). The entire genomic sequence was determined using an Illumina HiSeq 2000 instrument with the paired-end approach (average insert size, 350 bp) and a PacBio RS II (Pacific Biosciences) sequencing platform. Quality assessment was based on data passing the Illumina chastity filtering and then using FastQC ver. 0.10.0 for all reads. The Illumina paired-end sequencing yielded 4,630 Mb from 45,841,052 reads. The total length and total number of PacBio reads were 3,516 Mb and 238,713 reads. Those reads were assembled using SOAPdenovo ver. 2.04 (5) for Illumina and Canu ver. 1.5 (6) for the PacBio reads, and the two assemblies were merged using HaploMerger2 (7), all with default parameters. The draft genome of *D. pulvinata* strain 414-3 consisted of 71 scaffolds with a scaffold *N*_50_ value of 2,948,257 bp. The genome size was estimated to be 46,845,581 bp, and the G+C content was 57.6%. A total of 10,700 open reading frames were annotated using AUGUSTUS ver. 2.7 (8) based on gene models from Fusarium graminearum. All coding sequences were functionally annotated using BLAST (9) searches against the NCBI nonredundant (10), KEGG (11, 12), Clusters of Orthologous Groups (COG) (13), and Carbohydrate-Active EnZymes (CAZy) (14) databases. SignalP ver. 4.0 (15) was used to predict genes encoding secreted proteins. Future work will focus on identifying mycoparasitism-associated genes and exploring the molecular mechanisms to understand the parasitic capabilities of *D. pulvinata*.

### Data availability.

The nucleotide sequence of the whole-genome sequence of strain 414-3 has been deposited in DDBJ/EMBL/GenBank under the accession number BJKQ00000000. The DDBJ Sequence Read Archive (DRA) accession numbers are DRX170159 and DRX170160. This paper describes the first version of the genome.
